# Whole‐exome sequencing identified a novel homozygous *ASPH* frameshift variant causing Traboulsi syndrome in a Chinese family

**DOI:** 10.1002/mgg3.1553

**Published:** 2020-11-20

**Authors:** Cheng Lei, Ting Guo, Shuizi Ding, Liyan Liao, Hong Peng, Zhiping Tan, Hong Luo

**Affiliations:** ^1^ Department of Pulmonary and Critical Care Medicine the Second Xiangya Hospital Central South University Changsha China; ^2^ Research Unit of Respiratory Disease Central South University Changsha China; ^3^ Hunan Diagnosis and Treatment Center of Respiratory Disease Changsha China; ^4^ Department of Pathology the Second Xiangya Hospital Central South University Changsha China; ^5^ Clinical Center for Gene Diagnosis and Therapy Department of Cardiovascular Surgery The Second Xiangya Hospital of Central South University Changsha China

**Keywords:** ASPH, ectopia lentis, lung bullae, pneumothorax, traboulsi syndrome, ventricular septal defect

## Abstract

**Background:**

Traboulsi syndrome is a rare disorder characterized by ectopia lentis and facial dysmorphism (large beaked nose), which was only reported in 18 individuals to date. It is caused by homozygous/compound heterozygous variants in the aspartate/asparagine‐β‐hydroxylase (*ASPH*) gene, which hydroxylates the aspartic acid and asparagine in epidermal growth factor‐like domains of various proteins.

**Methods:**

Whole‐exome and Sanger sequencing were used to identify the disease‐causing gene of the patient in a consanguineous Chinese family. Domain analysis was applied to predict the impact of the variant on ASPH protein.

**Results:**

Through exome and Sanger sequencing, we identified a novel homozygous *ASPH* variant (NM_004318.4:c.1910del/NP_004309.2: p.(Asn637MetfsTer15)) in the patient, which may lead to blockage of the ASPH function through truncating the AspH oxygenase domain of the ASPH protein and/or nonsense‐mediated decay of the *ASPH* transcript. This is the first report of Traboulsi syndrome in a Chinese patient who was combined with ventricular septal defect, lung bullae, and recurrent spontaneous pneumothorax.

**Conclusion:**

Our results revealed the clinical characteristics of the first Chinese patient with Traboulsi syndrome. Additionally, our study expands the mutational spectrum of Traboulsi syndrome and provides information for clinical genetic counseling to this family.

## Graphical abstract

This is the first report of Traboulsi syndrome in a Chinses patient who was combined with recurrent spontaneous pneumothorax and ventricular septal defect. Our results revealed the clinical characteristics of the first Chinese patient with Traboulsi syndrome.

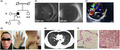

## 1 INTRODUCTION

Traboulsi syndrome (OMIM 601552), also known as FDLAB syndrome (Patel et al., 2014), is a rare syndrome characterized by facial dysmorphism, lens dislocation, anterior‐segment abnormalities, and spontaneous filtering blebs. It was first reported by Traboulsi and his colleagues in a multiplex consanguineous family from the Druze sect in Lebanon in 1995 (Shawaf et al., 1995). Two other unrelated Lebanese families were subsequently reported (Haddad et al., 2001; Mansour et al., 2013), and this syndrome was considered enriched in the Druze Lebanese population. Until 2014, Traboulsi and his colleagues undertook autozygosity mapping and whole‐exome sequencing in a Saudi female and two patients from previously reported Lebanese families, and they identified aspartate/asparagine‐β‐hydroxylase (*ASPH*) as the disease‐causing gene. In their study, the enzymatic function of ASPH was severely impaired by a truncating mutation (p.Ser589Glufs*18) in one patient and a missense (p.Arg735Trp) mutation in another two patients (Patel et al., 2014). *ASPH* (NCBI ID: 444) encodes aspartate/asparagine‐β‐hydroxylase (ASPH), which has been found to hydroxylate specific asparagine‐ and aspartate‐residues in epidermal growth factor (EGF)‐domain containing proteins, and it was reported to have a developmental role in the craniofacial region of engineered knockout mice model (Dinchuk et al., 2002). Eighteen cases of Traboulsi syndrome have been reported in Peru, India, Australia, and the United Kingdom (Abarca Barriga et al., 2018; Chandran et al., 2019; Haddad et al., 2001; Kulkarni et al., 2019; Mansour et al., 2013; Patel et al., 2014; Shanmugam et al., 2020; Shawaf et al., 1995; Siggs et al., 2019).

In this study, we performed whole‐exome and Sanger sequencing and identified a novel variant of *ASPH* in a patient who was born to consanguineous Chinese parents. He was finally diagnosed with Traboulsi syndrome. The patient was characterized by ectopia lentis, ventricular septal defect, lung bullae, and recurrent spontaneous pneumothorax while other family members were unaffected. He was previously misdiagnosed with Marfan syndrome due to the coexistence of lens dislocation (both eyes), tall stature, and thin body habitus. Our study expands the mutational spectrum of Traboulsi syndrome and provides information for clinical genetic counseling to this family.

## 2 METHODS

### 2.1 Ethical compliance

This study was approved by the Review Board of the Second Xiangya Hospital of Central South University in China in agreement with the Declaration of Helsinki. A Han Chinese consanguineous family participated in the study. Written informed consent was obtained from all participants.

### 2.2 Whole‐exome sequencing and bioinformatic pipeline

Peripheral blood samples (3–5 ml) from the proband (34‐year‐old, male) and his brother (37‐year‐old, unaffected) were obtained with informed consent, respectively. Genomic DNA was extracted using the DNeasy Blood & Tissue Kit (Qiagen, Valencia, CA) according to the manufacturer's instructions.

Whole‐exome capture and high‐throughput sequencing were performed by the Novogene Bioinformatics Institute (Beijing, China). Briefly, Genomic DNA was randomly fragmented to 180–280 bp using Covaris technology, and then, added adaptor at both ends of the fragments after end‐repairing and A‐tailing. The pooled DNA library was hybridized for exome capture by the Agilent SureSelect Human All ExonV6 Kit (Agilent). After polymerase chain reaction amplification, DNA sequencing was performed on the Illumina HiSeq 2500 system. The sequencing reads were aligned to the human reference genome (UCSC hg19; http://genome.ucsc.edu) using the Burrows‐Wheeler Alignment tool. Duplicate reads were removed using Picard, and variant calling and annotation were carried out using SAMtools and ANNOVAR.

### 2.3 Variant validation with Sanger sequencing

Sanger sequencing was used to validate the candidate variants identified by whole‐exome sequencing, and segregation analyses were performed in the family members. Primer pairs were designed using an online tool (PrimerQuest, IDT, https://eu.idtdna.com/pages/tools/primerquest). The primer sequences were designed as follows: forward, 5'‐GTCACTACCTATTGGAGCAAGAC‐3'; reverse, 5'‐GGCCAAAGGAAACAACCATTT‐3'. PCR products were sequenced by the ABI PRISM 3730 DNA Analyzer (Applera Corporation) using the ABI PRISM Big‐Dye Terminator Cycle Sequencing v.3.1 Ready Reaction Kit.

## 3 RESULTS

### 3.1 Clinical manifestation

**FIGURE 1:**
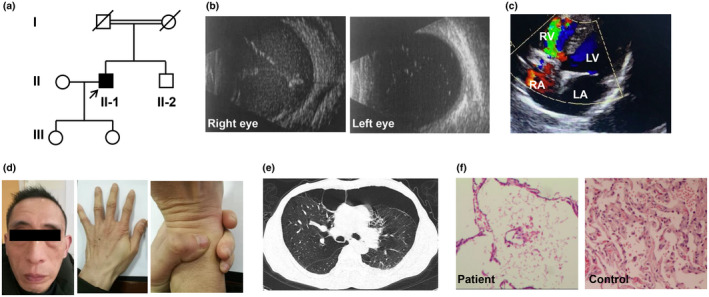
Pedigree of the family and clinical photographs. (a) Arrow showed the proband, all other people are unaffected. (b) Sonographic appearance of the patients’ aphakic eyes. (c) Ultrasound color Doppler 4‐chamber view of the patient showed ventricular septal defect. (d) The patient had an elongated face and relatively short fingers without wrist sign. (e) High‐resolution chest computed tomography showed diffuse emphysema, multiple bilateral subpleural blebs, bullae, and left lung pneumothorax. (f) Histological examination of the surgical lung specimen of the proband and the normal lung histology. LA, left atrium; LV, left ventricle; RA, right atrium; RV, right ventricle.

A 34‐year‐old Chinese male (II‐1) went to the emergency department of the Second Xiangya Hospital with a 3‐month history of recurrent shortness of breath that had been aggravated for 5 days without any identifiable trigger. The patient was a lean tall man, he was 186.0 cm tall and weighed 59.0 kg (body mass index 17.05 kg/m^2^). He had been clinically diagnosed with Marfan syndrome because of ectopia lentis (both eyes), tall stature, and thin body habitus 15 years ago. He was high myopia without other reported ocular defects, such as spontaneous filtering blebs, iris atrophy, retinal detachment, shallow anterior chambers, or closed iridocorneal angles. The lensectomy was performed for ectopia lentis in both eyes within 1 year after the initial diagnosis. After a comprehensive clinical assessment, we found the patient's parents are consanguineous (Figure 1a), which was inconsistent with the heredity mode of Marfan syndrome. His eyes were aphakic because the ectopic lenses had been removed 17 years ago (Figure 1b). Echocardiography indicated ventricular septal defect (Figure 1c), and pulmonary hypertension, but without aortic root complications such as aortic root dilatation or dissection which was necessary for clinical diagnosis of Marfan syndrome in the absence of family history and genetic testing (Loeys et al., 2010). He had relatively short fingers without wrist sign or thumb sign, and the patient had an elongated face but without other specific features (Figure 1d). High‐resolution chest computed tomography showed diffuse emphysema, multiple bilateral subpleural blebs, bullae, and left lung pneumothorax (Figure 1e). The bullae are predominately distributed in the bilateral upper lobes and left lower lobe (Figure S1). He also had a 10‐year history of smoking (about seven cigarettes per day), which may be related to the pulmonary emphysema in the lung computed tomography. He was diagnosed with left lung recurrent spontaneous pneumothorax, aphakic eye, and ventricular septal defect in admission. After the removal of pulmonary bulla, pleurodesis, and pleural closed drainage, the respiratory symptom was relieved. Histological examination of the surgical specimen (hematoxylin and eosin staining) showed the pulmonary alveoli dilated and fused into large capsular spaces, and pulmonary hemosiderosis was noticed in lung interstitial tissue (Figure 1f). One year later, secondary glaucoma and retinal detachment occurred in the right eye because the right eye was accidentally poked by a toothbrush. The patient recovered after vitrectomy and retinal laser photocoagulation of the right eye. No ocular, pulmonary, or cardiac defects were reported for the patient's parents, his brother, and his children.

### 3.2 Whole‐exome sequencing

Whole‐exome sequencing generated 10.96 GB data for the proband and 10.54 GB data for his brother (Table S1). The coverage for the target region was 99.90%. After alignment to reference human genome and variant calling, a total of 33576 INDELs (insertion/deletions) and 235033 SNVs (single nucleotide variants) were detected.

### 3.3 Variants filtering

**FIGURE 2:**
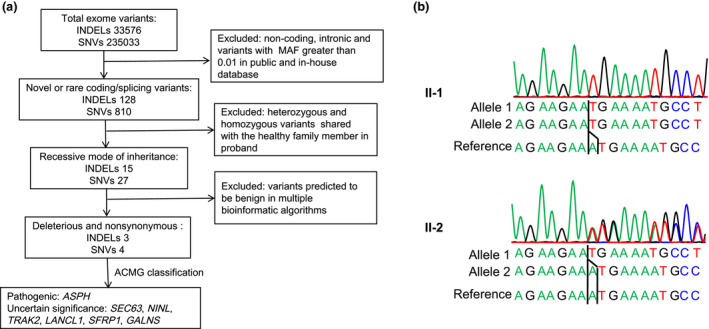
Exome sequencing filtering strategy (a) and chromatogram of the ASPH mutations (b). MAF, minor allele frequency.

The variant filtering process is illustrated in Figure 2a. Noncoding, intronic variants, and variants with a minor allele frequency of >1% in public data sets (1000 Genomes Project data set; NHLBI Exome Sequencing Project Exome Variant Server; ExAC, Exome Aggregation Consortium) or in‐house database of Novogene were excluded. Because the disorder was inherited through consanguineous marriage. Homozygous variants that were in accord with the recessive mode of inheritance were retained. The deleteriousness of variants was further explored by SIFT (https://sift.bii.a‐star.edu.sg), MutationTaster (http://www.mutationtaster.org), Polyphen‐2 (http://genetics.bwh.harvard.edu/pph2/), and CADD (https://cadd.gs.washington.edu, A score greater than 15 was consider damaging). Variants predicted to be benign in more than half of these algorithms were filtered.

After the filtering process, three INDELs and four SNVs were classified according to the guidelines for the interpretation of sequence variants by the American College of Medical Genetics and Genomics (ACMG; Richards et al., 2015). Only one INDEL mutation was classified as pathogenic (Table 1).

**TABLE 1:** The filtered variations of whole‐exome sequencing.

Gene	Variant type	Transcript ID	Transcript variant	Protein variant	SIFT	MutationTaster	Polyphen2_HVAR	CADD	ExAC MAF	ACMG	OMIM phenotype
*ASPH*	INDEL	NM_004318	c.1910delA	p.(N637 Mfs*15)	—	—	—	—	—	Pathogenic	AR; Traboulsi syndrome
*NINL*	INDEL	NM_025176	c.2873_2878del	p.(W958_P960delinsS)	—	—	—	—	—	Uncertain significance	—
*SEC63*	INDEL	NM_007214	c.340‐5_340‐4insGCC	—	—	—	—	—	—	Uncertain significance	AD; Polycystic liver disease 2
*TRAK2*	SNV	NM_015049	c.2070C>G	p.(S690R)	Damaging	Disease‐causing	Probably damaging	Damaging	—	Uncertain significance	—
*LANCL1*	SNV	NM_001136574	c.484C>T	p.(L162F)	Damaging	Disease‐causing	Probably damaging	Damaging	0.0006	Uncertain significance	—
*SFRP1*	SNV	NM_003012	c.692A>G	p.(K231R)	Tolerated	Disease‐causing	Benign	Damaging	0.0001	Uncertain significance	—
*GALNS*	SNV	NM_000512	c.576A>T	p.(E192D)	Tolerated	Disease‐causing	Possibly damaging	—	—	Uncertain significance	AR; Mucopolysaccharidosis IVA

Abbreviations: AD, autosomal dominant; AR, autosomal recessive; ExAC, the exome aggregation consortium; MAF, minor allele frequency; OMIM, Online Mendelian Inheritance in Man.

This novel variant was predicted to result in a frameshift mutation at codon 637 in exon 23 and premature termination of ASPH. It was not found in the in‐house database of Novogene, 1000 Genomes Project, ExAC, and gnomAD v2.1.1 data sets. Exome sequencing data indicated that this candidate disease‐causing variant of *ASPH* was homozygous in the patient and heterozygous in his brother.

### 3.4 Sanger sequencing for exon 23 of *ASPH*

Sanger sequencing indicated that a novel frameshift homozygous mutation (NM_004318.4:c.1910del/ NP_004309.2: p.(Asn637MetfsTer15)) of ASPH cosegregated with the patient (II‐1; Figure 2b). His brother (II‐2) was unaffected, and Sanger sequencing showed he was heterozygous for this variant.

### 3.5 The novel variant impaired the enzymatic function of ASPH

To delineate the influence of c.1910delA/ p.(Asn637MetfsTer15) for ASPH protein, we fetched the ASPH gene structure data from the Ensembl gene database (https://www.ensembl.org), protein domains information from the UniProtKB database (https://www.uniprot.org/uniprot/) and the literature (Pfeffer et al., 2019).

**FIGURE 3:**
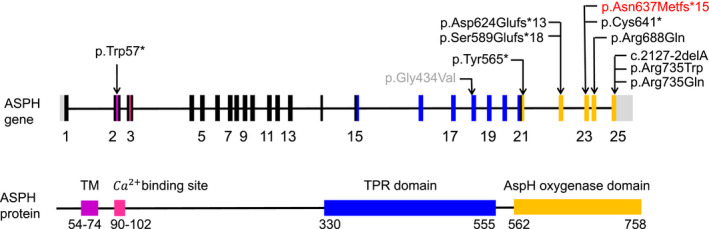
ASPH gene and protein structure and the reported disease‐causing mutations of ASPH. The up panel shows the exon of ASPH transcript NM_004318.4. The bottom panel shows the ASPH protein structure. Domains and their relative exons were filled using the same colors. Mutation in our study was highlight using red color. One reported single nucleotide variant associated with vesicoureteral reflux was highlight using gray color. ASPH, Aspartyl/asparaginyl beta‐hydroxylase; TM, transmembrane domain; TPR, tetratricopeptide repeat.

The transcript of *ASPH* (NM_004318.4) has 25 exons. Three SNVs (p.Arg735Gln, p.Arg735Trp, and p.Arg688Gln) are located in the AspH oxygenase domain and five frameshift variants that may truncate the AspH oxygenase domain have been reported to cause Traboulsi syndrome. Another SNV, p.Gly434Val is located to the tetratricopeptide repeat (TPR) domain was reported to cause vesicoureteral reflux, but whether it could cause Traboulsi syndrome remained unknown because the lens subluxation of the patient may be related to hyperhomocysteinemia, and no further information was provided for this patient (Vivante et al., 2017). In our study, the variant is located at the exon 23, which impaired the enzymatic function of ASPH through truncating the AspH oxygenase domain and/or nonsense‐mediated decay of the entire *ASPH* transcript (Figure 3).

## 4 DISCUSSION

In this study, we investigated the first Chinese consanguineous family with Traboulsi syndrome. The clinical manifestation of our patient included a nonspecific facial feature, ectopia lentis, left lung recurrent spontaneous pneumothorax, and ventricular septal defect which was different from previously reported. A novel homozygous *ASPH* mutation (NM_004318.4:c.1910del/ NP_004309.2: p.(Asn637MetfsTer15)) was identified in our patient by whole‐exome and Sanger sequencing.

Traboulsi syndrome is a rare autosomal recessive genetic disease with two prominent clinical manifestations, one is ectopia lentis, another is facial dysmorphism (large beaked nose; Abarca Barriga et al., 2018). In 2014, Traboulsi syndrome was found to be linked to *ASPH*, an enzyme that hydroxylates asparagine‐ and aspartate‐residues on epidermal growth factor (EGF) domains of proteins (Patel et al., 2014). The facial dysmorphism was consistent with the phenotype of the engineered knockout mice model, which showed a shortened snout. In situ hybridization revealed stronger *ASPH* expression in the limbs, snout, and eyes of the healthy developing mouse embryos. However, the eyes were not examined in any *ASPH* knockout animal model (Dinchuk et al., 2002; Patel et al., 2014). No other organs were reported to be involved.

ASPH is a nonheme ferrous iron and 2‐oxoglutarate oxygenase that localizes to the endoplasmic reticulum. It is a large protein consisted of 758 amino acids, which contains a transmembrane domain, a Ca^2+^‐binding site, a tetratricopeptide repeat, and an AspH oxygenase domain. ASPH can hydroxylate multiple proteins including coagulation factors (VII, IX, and X), protein C, complementation factors, thrombomodulin, low‐density lipoprotein receptor, and Notch ligands (Loenarz & Schofield, 2011). All these proteins have a consensus motif (CX[DN]4X[FY]XCXC) in EGF‐domain for asparagine‐ or aspartate‐hydroxylation (Pfeffer et al., 2019). ASPH consensus hydroxylation motif was identified in several lenticular phenotypes associated genes such as latent transforming growth factor beta‐binding protein‐2 (LTBP2) and fibrillin‐1 gene (FBN1) which were essential in microfibril and ciliary zonule development (Siggs et al., 2019). However, the biological importance of ASPH‐catalyzed hydroxylation in these proteins was undefined (Markolovic et al., 2015). Up to now, several *ASPH* variants have been reported to cause Traboulsi syndrome. All Traboulsi syndrome associated variants affected the AspH oxygenase domain that was essential for the substrates hydroxylation (Figure 3; Pfeffer et al., 2019). The variant p.Gly434Val is located to the tetratricopeptide repeat (TPR) domain was reported to cause vesicoureteral reflux, and the AspH oxygenase domain was unaffected (Vivante et al., 2017). In our study, the variant is located in the 23rd exon of *ASPH* gene, which may truncate the AspH oxygenase domain and/or lead to nonsense‐mediated decay of the *ASPH* transcript thus impaired the function of ASPH.

Our patient presented with ventricular septal defect, ectopia lentis in 19 year old, recurrent spontaneous pneumothorax in 34 year old. His facial feature was not specific, and it was also reported in a patient with Traboulsi syndrome from a UK family, which may be related to a different ethnic background (Kulkarni et al., 2019). To diagnose Traboulsi syndrome in patients without typical facial features, differential diagnoses should include syndromes that cause ectopia lentis such as Marfan syndrome and isolated ectopia lentis. In our patient, Traboulsi syndrome was confirmed through exome and Sanger sequencing after excluded deleterious mutations related to ectopia lentis including *ADAMTS10*, *ADAMTS17*, *ADAMTSL4*, *CBS*, *COL18A1*, *FBN1*, *LTBP2*, *PAX6*, and *VSX2* (Chandra & Charteris, 2014).

Marfan syndrome is a human autosomal dominant disease caused by loss‐of‐function variants in *FBN1*. The clinical features of Marfan syndrome include aortic root aneurysm/dissection, ectopia lentis, and findings in other organ systems such as skeleton, skin, and lungs (Loeys et al., 2010). In our study, ectopia lentis and spontaneous pneumothorax presented in our patient with Traboulsi syndrome are overlapped with Marfan syndrome. A previous study showed ASPH‐mediated hydroxylation of FBN1/LTBP2 may be associated with ectopia lentis (Siggs et al., 2019). Furthermore, FBN1 can bound TGFβ and prevent abnormal activation of TGFβ signaling in lung, aorta, and skeletal muscle, which is possibly responsible for many Marfanoid features including pneumothorax (Neptune et al., 2003; Sakai et al., 2016). Although the role of *FBN1* in Traboulsi syndrome was undefined, these studies suggested that the overlapped phenotypes may attribute to the molecular link between ASPH and FBN1.

However, it is noteworthy that whether ventricular septal defect and recurrent spontaneous pneumothorax are related to the *ASPH* mutation remained unknown, because only one patient was found in this consanguineous Chinese family. Furthermore, recurrent spontaneous pneumothorax occurred in our patient may also be attributed to his smoking history and low body mass index (Tschopp et al., 2015). Additional investigation is warranted to explore the mechanism underlying Traboulsi syndrome and to determine the phenotypic spectrum of Traboulsi syndrome in different ethnic people and in animal models.

In conclusion, we presented the clinical characteristics of a patient from a consanguineous Chinese family with ventricular septal defect, recurrent spontaneous pneumothorax, and ectopia lentis. By using whole‐exome and Sanger sequencing, a novel mutation in *ASPH* associated with Traboulsi syndrome was identified. Sequence analysis indicated that this novel variant may truncate the AspH oxygenase domain and/or lead to nonsense‐mediated decay of the *ASPH* transcript. This is the first report of Traboulsi syndrome in a Chinese patient, and our study expands the spectrum of *ASPH* variants and provides information for clinical genetic counseling to this family.

## CONFLICT OF INTEREST

The authors declare no conflict of interest.

## AUTHORS’ CONTRIBUTION

H.L and Z‐p.T conceived and designed the experiments. C.L, T.G, and S‐z.D perform the experiments, analyzed the data. L‐y.L and H.P collected samples and the clinical data. C.L, T.G, and S‐z.D wrote the manuscript. All authors critically reviewed and approved the final version of the manuscript.

## Supporting information

Supplementary Material

## Data Availability

The data that support the findings of this study are available from the corresponding author upon reasonable request.
