# Supplementary material for: Whole‐exome sequencing identified a novel homozygous ASPH frameshift variant causing Traboulsi syndrome in a Chinese family
Source: Mol Genet Genomic Med. 2020 Nov 20;9(1):e1553. doi: 10.1002/mgg3.1553 (PMC7963421; doi:10.1002/mgg3.1553)
Supplement: Supplementary file 1 — Supplementary Material [file MGG3-9-e1553-s001.pdf]

Supplemental Information

Supplementary Table S1 Summary of SNVs and INDELs for exome captured samples

	II-1	II-2
**Exome Capture Statistics**		
Raw data(G)	10.96	10.54
Total reads (Clean reads)	72733980	69911118
Mapped reads	72541203	69746282
Total_effective_mapped(Mb)	10802.03	10364.52
Effective_sequences_on_target_region(Mb)	6510.78	6551.22
Fraction_of_effective_bases_on_target_region	60.30%	63.20%
Average_sequencing_depth_on_target_region	107.69	108.36
Base_covered_on_target	60383910	60385692
Coverage_of_target_region	99.90%	99.90%
Fraction of target covered＞=4×（%）	99.80%	99.80%
Fraction of target covered＞=10×（%）	99.40%	99.50%
Fraction of target covered＞=20×（%）	98.40%	98.40%
**Variants Statistics**		
Total number of SNVs	185215	166714
Missense	10540	10519
synonymous SNV	11486	11442
Splice site	2472	2479
Hom	89893	79472
Het	95322	87242
Total number of indels	24222	22533
frameshift_deletion	68	70
frameshift_insertion	46	50
nonframeshift_deletion	193	195
nonframeshift_insertion	188	170
stopgain	4	4
Hom	11541	10651
Het	12681	11882

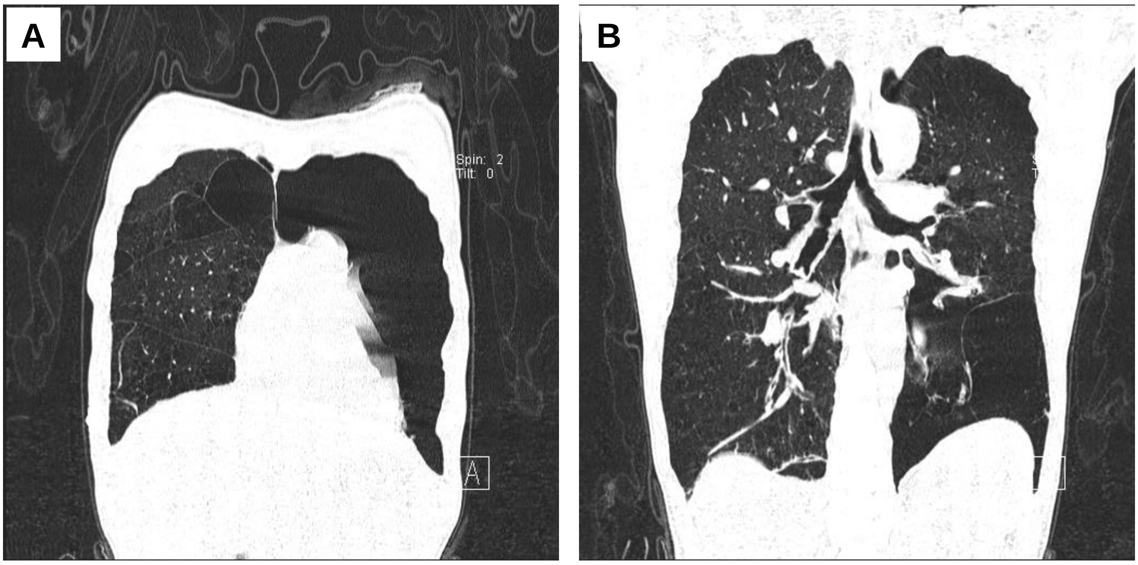

Supplementary Figure S1 Coronal view of high-resolution chest computed tomography demonstrating the bullae are predominately distributed in the bilateral upper lobes (A) and left lower lobe(B).
